# The Symmetrical Wave Pattern of Base-Pair Substitution Rates across the Escherichia coli Chromosome Has Multiple Causes

**DOI:** 10.1128/mBio.01226-19

**Published:** 2019-07-02

**Authors:** Brittany A. Niccum, Heewook Lee, Wazim MohammedIsmail, Haixu Tang, Patricia L. Foster

**Affiliations:** a Department of Biology, Indiana University, Bloomington, Indiana, USA; b School of Informatics, Computing, and Engineering, Indiana University, Bloomington, Indiana, USA; University of British Columbia; Brandeis University; University of Pittsburgh

**Keywords:** *Escherichia coli*, mismatch repair, mutation accumulation, mutation rate, nucleoid-associated proteins, nucleoside triphosphate pools, proofreading, replication timing, whole-genome sequencing

## Abstract

Mutation accumulation experiments followed by whole-genome sequencing have revealed that, for several bacterial species, the rate of base-pair substitutions (BPSs) is not constant across the chromosome but varies in a wave-like pattern that is symmetrical about the origin of replication. The experiments reported here demonstrated that, in Escherichia coli, several interacting factors determine the wave. The origin is a major driver of BPS rates. When it is relocated, the BPS rates in a 1,000-kb region surrounding the new origin reproduce the pattern that surrounds the normal origin. However, the pattern across distant regions of the chromosome is unaltered and thus must be determined by other factors. Increasing the deoxynucleoside triphosphate (dNTP) concentration shifts the wave pattern away from the origin, supporting the hypothesis that fluctuations in dNTP pools coincident with replication firing contribute to the variations in the mutation rate. The nucleoid binding proteins (HU and Fis) and the terminus organizing protein (MatP) are also major factors. These proteins alter the three-dimensional structure of the DNA, and results suggest that mutation rates increase when highly structured DNA is replicated. Biases in error correction by proofreading and mismatch repair, both of which may be responsive to dNTP concentrations and DNA structure, also are major determinants of the wave pattern. These factors should apply to most bacterial and, possibly, eukaryotic genomes and suggest that different areas of the genome evolve at different rates.

## Abstract (precis)

It has been found in several species of bacteria that the rate at which single base pairs are mutated is not constant across the genome but varies in a wave-like pattern that is symmetrical about the origin of replication. Using Escherichia coli as our model system, we show that this pattern is the result of several interconnected factors. First, the timing and progression of replication are important in determining the wave pattern. Second, the three-dimensional structure of the DNA is also a factor, and the results suggest that mutation rates increase when highly structured DNA is replicated. Finally, biases in error correction, which may be responsive both to the progression of DNA synthesis and to DNA structure, are major determinants of the wave pattern. These factors should apply to most bacterial and, possibly, eukaryotic genomes and suggest that different areas of the genome evolve at different rates.

## INTRODUCTION

Recent studies of mutations accumulated nonselectively across bacterial chromosomes revealed that rates of base-pair substitutions (BPSs) vary 2-fold to 4-fold in a wave-like pattern that is mirrored in the two independently replicating halves of the chromosome. These symmetrical patterns have been observed in mismatch repair (MMR)-defective strains of Escherichia coli (1), Vibrio fischeri, V. cholerae (2–4), Pseudomonas fluorescens (5), and P. aeruginosa (6). Such variations in mutation rates may affect the pace at which genes in different regions of the chromosome evolve and may exert selective pressure on gene placement. Yet the causes of this variation are not known.

The fidelity of DNA replication, which, in E. coli, is about 1 mistake in 1,000 generations (7), is determined by the intrinsic accuracy of the DNA polymerase plus error correction by proofreading and MMR (8, 9). In E. coli, proofreading is performed by epsilon, a subunit of the DNA polymerase III holoenzyme. If the polymerase inserts an incorrect base, the 5′ to 3′ exonuclease activity of epsilon degrades a few bases of the new strand and polymerase then resynthesizes it, improving the accuracy of DNA synthesis about 4,000-fold (10). MMR is initiated by MutS and MutL, which recognize a mismatch and find a nearby GATC site. The As in GATC sites in E. coli are methylated by the Dam methylase; because methylation lags behind replication, unmethylated As identify the “new” and, presumably, error-containing DNA strand. MutH is recruited by MutSL and activated to nick the unmethylated strand, which is then degraded past the mismatch. Pol III then resynthesizes the strand. MMR improves the accuracy of DNA replication 100-fold to 200-fold (11).

In our previous study of the BPS density pattern in MMR-defective E. coli (1), we correlated BPS rates to the chromosomal sites that are affected by two nucleoid-associated proteins (NAPs), HU and Fis. We suggested that when the replication fork encounters regions of the chromosome with high superhelical density due to the binding of these NAPs, the mutation rate increases. An alternative explanation ties mutation rates to replication timing (2, 3). An intriguing hypothesis is that mutation rates vary in concert with fluctuations in deoxynucleoside triphosphate (dNTP) pools when the replication origin fires repeatedly during rapid growth (2).

In the work whose results are presented here, we investigated further the causes of the wave-like pattern of BPS rates. MMR-defective strains of E. coli additionally defective for other activities were used in mutation accumulation (MA) experiments followed by whole-genome sequencing (WGS). We also investigated the effects of different growth conditions on BPS rates. We conclude that the BPS density pattern does not have a single cause but is the result of the activity of several factors affecting DNA replication, repair, and chromosome structure. In addition, we report that a MMR-defective Bacillus subtilis strain also has a wave-like BPS rate pattern that is symmetrical about the origin of replication.

## RESULTS

### The base-pair substitution (BPS) pattern in mismatch repair-defective strains.

**FIG 1:**
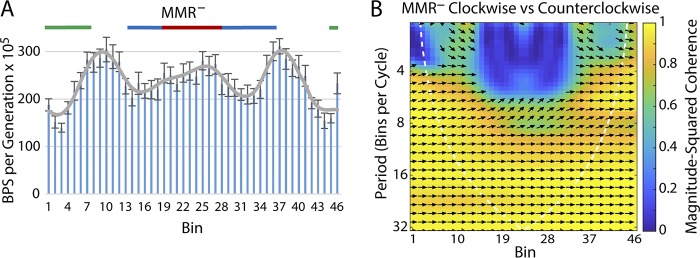
The BPS rate pattern of MMR-defective strains. The BPS data from 10 experiments performed with MMR-defective strains were collected into 100-kb bins starting at the origin of replication shown on the left (bin 1) and continuing clockwise around the chromosome back to the origin shown on the right (bin 46). (A) Bars represent the mean BPS rate in each bin; error bars represent 95% CL of the mean. The gray line represents the Daubechies wavelet transform of the binned data. The chromosomal macrodomains (MDs) are indicated at the top of the plot as follows: green, Ori MD; blue, right and left MD; red, Ter MD. (B) Plot of the wavelet coherence between the binned BPS data from the MMR-defective strains taken in the clockwise and counterclockwise directions around the chromosome. The colors indicate the magnitude-squared coherence, a measure of the correlation between the data sets, according to the scale on the right. The dotted line gives the “cone of influence” within which the results are free of artifactual edge effects. Arrows indicate the phase lag between the two data sets; arrows pointing right indicate in phase, arrows pointing left indicate 180° out of phase, and arrows pointing in other directions indicate the various degrees between.

We demonstrated in a previous paper (1) that the rates at which BPSs accumulated across the chromosome during an MA experiment with a MMR-defective strain fell into a wave-like pattern symmetrical about OriC, the origin of replication. We have since performed nine additional MA experiments with MMR mutant strains, yielding a total of 30,061 BPSs (11). As shown in Fig. 1A, the wave-like pattern of BPS rates was reproducible among the 10 experiments. Also, the pattern does not exactly match that corresponding to the noninteracting chromosomal macrodomains (MDs) as defined by Valens et al. (12). In particular, the wave pattern of BPSs is symmetrical about the origin whereas the Ori MD is not. In the analyses described below, the combined data from the 10 experiments performed with MMR-defective strains are used as the standard to which the results obtained in other genetic backgrounds are compared.

Following the analysis of Dillon et al. (2), we computed the wavelet coherence of the MMR^–^ data taken in the clockwise and counterclockwise directions around the chromosome. Panel B of Fig. 1 shows that, except for some asymmetry at the midpoint, the wave is symmetrical across the chromosome. The coherence is greatest between 800 and 1,600 kb per cycle (i.e., 8 to 16 bins per cycle), which is similar to that found by Dillon et al. (2). We then computed the wavelet coherence of the collected MMR^–^ data against each of the experimental results presented below.

The spectrum of BPSs in the MMR-defective strains is dominated by A:T transitions at 5′NAC3′/3′NTG5′ sites (11). To test if the mutational density pattern was simply due to the distribution of these sites, we removed from the data all A:T transitions at 5′NAC3′/3′NTG5′ sites. As given in Tables 1 and 2, the remaining BPSs fell into the same wave pattern. Thus, the variation in BPS rates must be reflective of regions of the chromosome and not of the distribution of the hot spot triplets.

**TABLE 1:** Pearson's correlation ρ for each strain versus MMR^−^ strains

Strain(s)	Description	Whole chromosome (bins 1 to 46)		Right replichore (bins 1 to 23)		Left replichore (bins 46 to 24)		Origin (bins 1 to 13 and 46 to 34)		Terminus (bins 14 to 33)	
		ρ	*P*^*a*^	ρ	*P*^*a*^	ρ	*P*^*a*^	ρ	*P*^*a*^	ρ	*P*^*a*^
Collective^*b*^	MMR^−^										
Collective	MMR^−^ minus A:T→G:C at 5′NAC3′/3′NTG5′ sites	0.94	<0.001	0.93	<0.001	0.96	<0.001	0.97	<0.001	0.71	0.004
PFM421	Δ*rnhA* Δ*mutL*	0.74	<0.001	0.63	0.002	0.84	<0.001	0.81	<0.001	0.47	0.075
PFM669	Δ*mutL* (AB1157)	0.59	<0.001	0.40	0.064	0.75	<0.001	0.71	<0.001	0.45	0.079
PFM430/431	*oriC^+^ oriZ^+^* Δ*mutL* (AB1157)	0.75	<0.001	0.71	<0.001	0.81	<0.001	0.83	<0.001	0.46	0.075
PFM426	Δ*oriC oriZ^+^* Δ*mutL* (AB1157)	0.39	0.008	0.21	0.34	0.62	0.003	0.40	0.044	0.41	0.11
PFM533/534	Δ*seqA* Δ*mutL*	0.49	0.001	0.71	<0.001	0.27	0.23	0.53	0.006	0.38	0.14
PFM5^*b*^	Δ*mutL* on minimal medium	0.50	0.001	0.40	0.069	0.61	0.003	0.57	0.003	0.58	0.029
PFM343^*b*^	Δ*mutS* on minimal medium	0.18	0.22	0.18	0.41	0.18	0.41	0.21	0.31	0.22	0.38
PFM343^*b*^	Δ*mutS* on diluted LB	0.84	<0.001	0.84	<0.001	0.85	<0.001	0.87	<0.001	0.71	0.004
PFM343^*b*^	Δ*mutS* on supplemented minimal medium	0.81	<0.001	0.82	<0.001	0.81	<0.001	0.83	<0.001	0.65	0.008
PFM342^*b*^	Δ*mutS* at low temperature	0.57	<0.001	0.60	0.003	0.53	0.013	0.69	<0.001	−0.02	0.95
PFM799	Δ*nrdR* Δ*mutL*	0.49	0.001	0.58	0.005	0.39	0.080	0.69	<0.001	−0.31	0.24
PFM677	Δ*rep* Δ*mutL*	0.48	0.001	0.49	0.022	0.48	0.027	0.62	0.001	−0.12	0.67
PFM256	Δ*tus* Δ*mutL*	0.82	<0.001	0.79	<0.001	0.85	<0.001	0.87	<0.001	0.70	0.004
PFM257	Δ*matP* Δ*mutL*	0.40	0.007	0.60	0.004	0.21	0.34	0.70	<0.001	−0.47	0.075
PFM422	Δ*recA* Δ*mutL*	0.71	<0.001	0.70	<0.001	0.73	<0.001	0.85	<0.001	0.06	0.84
PFM424	Δ*recA* Δ*mutS*	0.80	<0.001	0.78	<0.001	0.83	<0.001	0.91	<0.001	0.44	0.082
PFM456	Δ*recB* Δ*mutL*	0.82	<0.001	0.87	<0.001	0.79	<0.001	0.89	<0.001	0.39	0.12
PFM118	Δ*umuDC* Δ*dinB* Δ*mutL*	0.59	<0.001	0.62	0.003	0.55	0.010	0.61	0.001	0.69	0.004
PFM120	*lexA3* Δ*sulA* Δ*mutL*	0.82	<0.001	0.82	<0.001	0.83	<0.001	0.83	<0.001	0.80	0.001
PFM259	Δ*hupB* Δ*mutL*	0.73	<0.001	0.70	<0.001	0.77	<0.001	0.81	<0.001	0.57	0.031
PFM258	Δ*hupA* Δ*mutL*	0.49	0.001	0.43	0.053	0.58	0.005	0.67	<0.001	−0.23	0.38
PFM317/318	Δ*fis* Δ*mutL*	0.51	<0.001	0.61	0.003	0.42	0.060	0.67	<0.001	−0.30	0.25
PFM482	Δ*fis* Δ*mutS*	0.49	0.001	0.31	0.15	0.62	0.003	0.65	<0.001	−0.28	0.27
PFM741	Δ*hns* Δ*mutL*	0.81	<0.001	0.81	<0.001	0.81	<0.001	0.88	<0.001	0.46	0.075
PFM713	Δ*dps* Δ*mutL*	0.80	<0.001	0.84	<0.001	0.78	<0.001	0.89	<0.001	0.47	0.075
PFM163^*c*^	*mutD5*	0.76	<0.001	0.79	<0.001	0.74	<0.001	0.82	<0.001	0.78	0.001
PFM165/397/399^*c*^	*mutD5* Δ*mutL*	0.64	<0.001	0.76	<0.001	0.52	0.014	0.86	<0.001	−0.46	0.075
Collective^*b*^	Wild type	0.36	0.014	0.41	0.061	0.31	0.17	0.37	0.065	0.51	0.060
Collective^*d*^	Bacillus subtilis *mutS*::Tn*10*	0.52	<0.001	0.63	0.003	0.40	0.068	0.74	<0.001	−0.52	0.055

a *P* values were adjusted for multiple comparisons using the Benjamini-Hochberg method (56) with the false-discovery rate set at 25%.

b Data are from reference 11.

c Data are from reference 10.

d Data were combined from four MA experiments performed with DK2140 (NCIB3610 *mutS*::Tn*1*0), DK2141 (NCIB3610 *mutS*::Tn*10*), DK2142 (NCIB3610 *mutS*::Tn*10* Δ*comI*), and DK2143 (NCIB3610 *mutS*::Tn*10* Δ*com*I). There were no differences in mutation rates or spectra among these strains.

**TABLE 2:** Pearson's correlation ρ of right versus left replichore for each strain

Strain	Description	Whole chromosome (bins 1 to 23 and 46 to 24)		Origin (bins 1 to 13 and 46 to 34)		Terminus (bins 14 to 23 and 33 to 24)	
		ρ	*P*^*a*^	ρ	*P*^*a*^	ρ	*P*^*a*^
Collective^*b*^	MMR^−^	0.91	<0.001	0.95	<0.001	0.66	0.23
Collective	MMR^−^ minus A:T→G:C at 5′NAC3′/3′NTG5′ sites	0.89	<0.001	0.94	<0.001	0.42	0.60
PFM421	Δ*rnhA* Δ*mutL*	0.49	0.026	0.58	0.051	0.24	0.82
PFM669	Δ*mutL* (AB1157)	0.52	0.018	0.64	0.026	0.29	0.77
PFM430/431	*oriC^+^ oriZ^+^* Δ*mutL* (AB1157)	0.60	0.005	0.80	0.003	0.19	0.84
PFM426	Δ*oriC oriZ^+^* Δ*mutL* (AB1157)	0.28	0.22	0.33	0.31	−0.06	0.91
PFM533/534	Δ*seqA* Δ*mutL*	0.41	0.062	0.53	0.079	0.32	0.71
PFM5^*b*^	Δ*mutL* on minimal medium	0.27	0.22	0.19	0.53	0.05	0.91
PFM343^*b*^	Δ*mutS* on minimal medium	−0.02	0.93	0.32	0.31	−0.53	0.39
PFM343^*b*^	Δ*mutS* on diluted LB	0.87	<0.001	0.93	<0.001	0.66	0.23
PFM343^*b*^	Δ*mutS* on supplemented minimal medium	0.74	<0.001	0.79	0.003	0.54	0.39
PFM342^*b*^	Δ*mutS* at low temperature	0.84	<0.001	0.89	<0.001	0.73	0.19
PFM799	Δ*nrdR* Δ*mutL*	0.79	<0.001	0.78	0.003	0.84	0.07
PFM677	Δ*rep* Δ*mutL*	0.43	0.053	0.58	0.051	−0.16	0.84
PFM256	Δ*tus* Δ*mutL*	0.70	0.001	0.88	<0.001	0.26	0.81
PFM257	Δ*matP* Δ*mutL*	0.45	0.042	0.40	0.20	0.72	0.19
PFM422	Δ*recA* Δ*mutL*	0.66	0.001	0.81	0.003	0.11	0.88
PFM424	Δ*recA* Δ*mutS*	0.64	0.002	0.80	0.003	0.07	0.91
PFM456	Δ*recB* Δ*mutL*	0.67	0.001	0.78	0.003	0.03	0.94
PFM118	Δ*umuDC* Δ*dinB* Δ*mutL*	0.39	0.075	0.68	0.018	−0.13	0.87
PFM120	*lexA3* Δ*sulA* Δ*mutL*	0.51	0.019	0.57	0.055	0.42	0.60
PFM259	Δ*hupB* Δ*mutL*	0.64	0.002	0.78	0.003	0.35	0.65
PFM258	Δ*hupA* Δ*mutL*	0.54	0.014	0.69	0.015	0.15	0.84
PFM317/318	Δ*fis* Δ*mutL*	0.61	0.004	0.67	0.019	0.58	0.34
PFM482	Δ*fis* Δ*mutS*	0.34	0.13	0.49	0.108	0.19	0.84
PFM741	Δ*hns* Δ*mutL*	0.81	<0.001	0.85	0.001	0.60	0.33
PFM713	Δ*dps* Δ*mutL*	0.52	0.017	0.77	0.004	−0.20	0.84
PFM163^*c*^	*mutD5*	0.75	<0.001	0.77	0.004	0.52	0.39
PFM165/397/399^*c*^	*mutD5* Δ*mutL*	0.78	<0.001	0.88	<0.001	0.39	0.62
Collective^*b*^	Wild type	0.17	0.46	0.24	0.45	0.17	0.84
Collective^*d*^	Bacillus subtilis *mutS*::Tn*10*	0.73	<0.001	0.87	<0.001	0.37	0.65

a *P* values were adjusted for multiple comparisons using the Benjamini-Hochberg method (56) with the false-discovery rate set at 25%.

b Data are from reference 11.

c Data are from reference 10.

d Data were combined from four MA experiments performed with DK2140 (NCIB3610 *mutS*::Tn*1*0), DK2141 (NCIB3610 *mutS*::Tn*10*), DK2142 (NCIB3610 *mutS*::Tn*10* Δ*comI*), and DK2143 (NCIB3610 *mutS*::Tn*10* Δ*com*I). There were no differences in mutation rates or spectra among these strains.

### Transcription.

**FIG 2:**
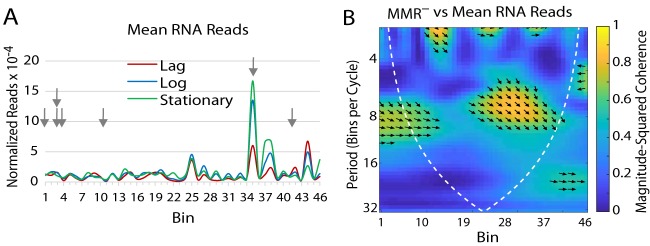
Comparison of the pattern of BPS rates to transcription levels. (A) The Daubechies wavelet transform of the binned reads from RNA-Seq samples taken during lag, log, and stationary-growth phases of Δ*mutL* mutant strain PFM144. The data used for the wavelet transforms represent means of results from three biological replicates. Arrows indicate the positions of the rRNA operons, but the reads from the ribosomal genes in those operons were not included in the plot (see Materials and Methods). (B) Plot of the wavelet coherence between the BPS data from the MMR-defective strains and the mean binned reads from RNA-Seq samples taken during lag, log, and stationary-growth phases of the Δ*mutL* mutant strain (see the legend to Fig. 1B for further explanation of the coherence plot.).

One obvious hypothesis is that the mutational pattern reflects transcriptional patterns. To test this hypothesis, we quantitated the RNA transcript levels in a Δ*mutL* mutant strain in the lag, exponential, and stationary phases of growth; the results should be representative of the cells growing in colonies during our MA experiments. To compare these results to the BPS rate pattern, we binned the transcriptome sequencing (RNA-Seq) results into the same bins used for mutational data. As shown in Fig. 2, there was no similarity in the two data sets. These data are further discussed in Text S1 in the supplemental material.

10.1128/mBio.01226-19.1 TEXT S1 Details regarding the RNA-Seq results (see Fig. 2). Download Text S1, DOCX file, 0.01 MB. Copyright © 2019 Niccum et al. 2019 Niccum et al. This content is distributed under the terms of the Creative Commons Attribution 4.0 International license.

### Replication initiation.

Over many experiments performed in different genetic backgrounds, the BPS pattern around OriC was consistent. In both replichores, the BPS rates declined to a minimum about 300 kb from OriC and then increased to a maximum about 1,000 kb from the origin. Mutation rates then fell again and reached a minimum about 3/5 of the distance toward the terminus.

We tested whether replication initiation was responsible for this pattern by performing MA experiments with strains with errant replication start sites. The *rnhA* gene encodes RNase H1, which degrades RNA-DNA hybrids; in its absence, persistent R-loops initiate aberrant DNA replication that can disrupt normal fork migration (13, 14). However, the BPS pattern of a Δ*rnhA* Δ*mutL* mutant strain showed little difference from that of the Δ*mutL* mutant strain (Table 1), indicating that these aberrant replication initiations do not influence where BPSs occur, at least not when a powerful *oriC* gene is present.

**FIG 3:**
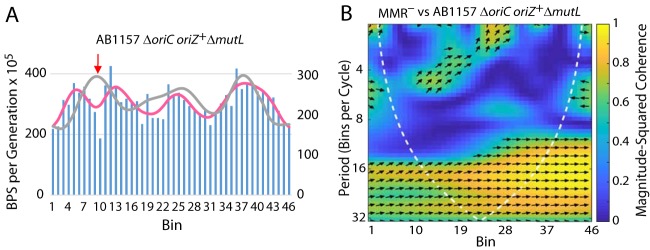
The effect on the BPS rate pattern of relocating OriC. (A) Bars represent the BPS rate in each bin for the Δ*oriC oriZ*^+^ Δ*mutL* mutant strain (PFM426), and the pink line represents the Daubechies wavelet transform of the binned data (scale on left axis). The gray line represents the Daubechies wavelet transform of the binned data from the 10 MMR^−^ strains (scale on right axis; adjusted to bring the wavelets together for comparison). (B) Plot of the wavelet coherences between the data from the Δ*oriC oriZ*^+^ Δ*mutL* mutant strain and the data from the 10 MMR^−^ strains. (See the legend to Fig. 1 for further explanations of the bar charts and the coherence plots.) The arrow in panel A indicates the position of *oriZ*^+^.

We also performed MA experiments with strains with *oriC* moved to the midpoint of the right replichore, where it is called *oriZ* (15). These strains are derived from E. coli K-12 strain AB1157, instead of the ancestor of our MA strains, MG1655. As a control, we tested an AB1157 Δ*mutL* mutant strain and found that its BPS rate pattern was similar to that of our MMR-defective strains, indicating that the pattern is intrinsic to E. coli K-12 and is not specific to substrain MG1655 (Table 1). We performed MA experiments on Δ*mutL* derivatives of a strain with both *oriZ* and *oriC* (strain WX320 Δ*mutL*) and one with only *oriZ* (strain WX340 Δ*mutL*). The strain with both origins had a BPS pattern similar to those seen with the MMR^–^ strains (Table 1), suggesting that, under our conditions, firing of *oriZ* could not overcome the influence of *oriC*. However, the strain containing only *oriZ* showed a decrease in the BPS rates in the 200-kb area surrounding the new origin, similarly to that normally observed about *oriC* (Tables 1 and 2; see also Fig. 3).

We also performed MA experiments with a strain deleted for SeqA, which controls replication initiation (Tables 1 and 2; see also Fig. S1A and B in the supplemental material). These results are further discussed in Text S2 in the supplemental material.

10.1128/mBio.01226-19.2 TEXT S2 Further discussion of the SeqA results (see Fig. S1A and B in the supplemental material). Download Text S2, DOCX file, 0.01 MB. Copyright © 2019 Niccum et al. 2019 Niccum et al. This content is distributed under the terms of the Creative Commons Attribution 4.0 International license.

10.1128/mBio.01226-19.6 FIG S1 Effect of loss of SeqA, RecA, and RecB on the BPS rate pattern. (A, C, and E) BPS data were collected into 100-kb bins starting at the origin of replication on the left (bin 1) and continuing clockwise around the chromosome back to the origin on the right (bin 46). Bars represent the BPS rate in each bin accumulated in the tested strain; the pink lines represent the Daubechies wavelet transform of the binned data (scale on left axis). The gray lines represent the Daubechies wavelet transform of the binned data from 10 experiments performed with MMR-defective strains (scale on right axis; adjusted to bring the wavelets together for comparison). (B, D, and F) Plots of the wavelet coherences between the data from the tested strain and the data from the 10 MMR^−^ strains. The colors correspond to the magnitude-squared level of coherence, a measure of the correlation between the data sets, according to the scale on the right. The dotted line indicates the “cone of influence” within which the results are free of artifactual edge effects. Arrows indicate the phase lag between the two data sets; arrows pointing right indicate in phase, arrows pointing left indicate 180° out of phase, and arrows pointing in other directions indicate the various degrees between. Strains: panels A and B, Δ*seqA* Δ*mutL* mutant strain PFM533/534; panels C and D, Δ*recA* Δ*mutL* mutant strain PFM422; panels E and F, Δ*recB* Δ*mutL* mutant strain PFM456. Download FIG S1, EPS file, 2.6 MB. Copyright © 2019 Niccum et al. 2019 Niccum et al. This content is distributed under the terms of the Creative Commons Attribution 4.0 International license.

### Replication progression.

Dillon et al. (2) hypothesized that the periodic variation in mutation rates across the chromosome was due to systematic fluctuations in dNTP levels as OriC repeatedly fires during rapid cell growth. High levels of dNTPs increase the probability of misincorporation, and thus the BPS rate, whereas low levels of dNTPs slow replication and improve fidelity (16, 17). The hypothesis of Dillon et al. can be tested by examining the wave pattern from several experiments that we have already published (11).

**FIG 4:**
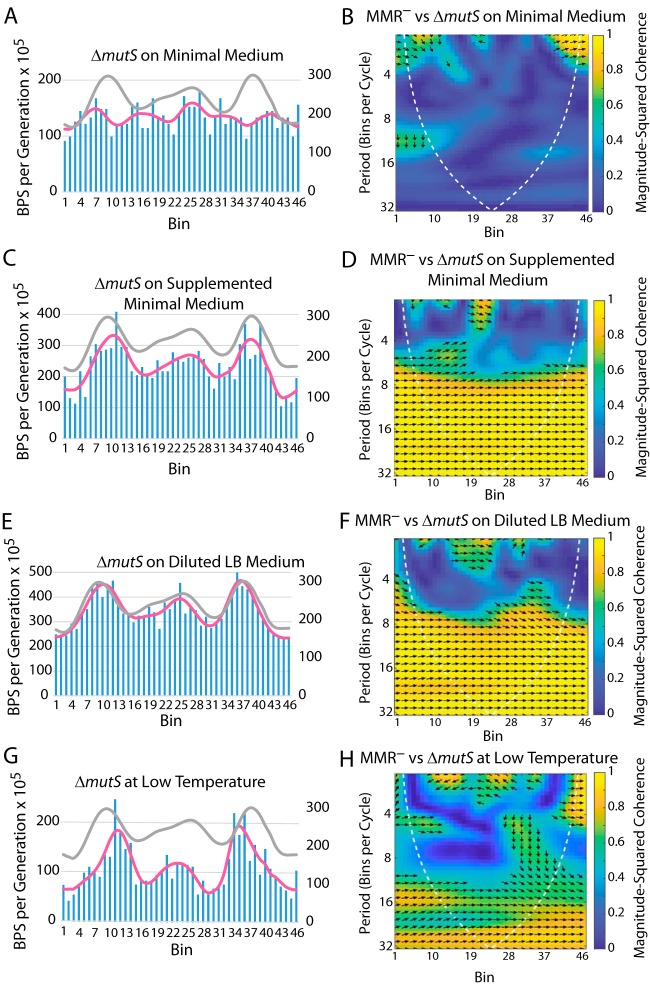
The effects on the BPS rate pattern of growing the cells on different media and at different temperatures. (A, C, E, and G) Bars represent the BPS rate in each bin for the tested strain; the pink lines represent the Daubechies wavelet transform of the binned data (scale on left axis). The gray lines represent the Daubechies wavelet transform of the binned data from the 10 MMR^−^ strains (scale on right axis; adjusted to bring the wavelets together for comparison). (B, D, F, and H) Plots of the wavelet coherences between the data from the tested strain and the data from the 10 MMR^−^ strains. (See the legend to Fig. 1 for further explanations of the bar charts and of the coherence plots.) The cells grew at the normal rate in the experiments represented in plots C and D, whereas they grew at about half the normal rate in the other plots. Strains: panels A to F, Δ*mutS* mutant strain PFM343; panels G and H, Δ*mutS* mutant strain PFM342.

The number of replicating chromosomes is a positive function of the cell’s growth rate (18). When a Δ*mutS* or a Δ*mutL* mutant strain was grown on glucose minimal medium, which reduced the growth rate about 2-fold relative to that on LB, the BPS rate pattern became chaotic (Tables 1 and 2; see also Fig. 4A and B). Supplementing the minimal medium with just enough LB to increase the growth rate to normal restored the wave-like BPS rate pattern (Tables 1 and 2; see also Fig. 4C and D). Growing the cells on diluted LB, on which the growth rate was the same as on minimal glucose medium, also preserved the wave-like BPS pattern (Tables 1 and 2; see also Fig. 4E and F). When the cells were grown at a low temperature that also reduced the growth rate 2-fold, the overall shape of the BPS pattern was retained, but the peaks in BPS rates that normally occurred 1,000 kb from the origin were shifted about 200 kb further from OriC (Fig. 4G and H). In addition, the magnitude of the BPS rate fluctuations across the chromosome was doubled. Thus, it appears that growth rate is not the only determinant of the BPS pattern but that other factors, such as the composition of the growth medium, are important.

**FIG 5:**
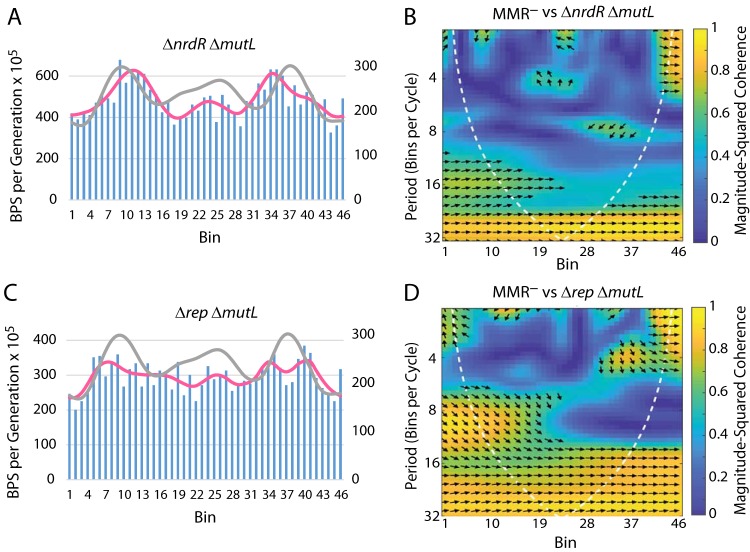
The effects on the BPS rate pattern of dysregulation of dNTP levels and loss of Rep, the auxiliary replication helicase. (A and C) Bars represent the BPS rate in each bin for the tested strain; the pink lines represent the Daubechies wavelet transform of the binned data (scale on left axis). The gray lines represent the Daubechies wavelet transform of the binned data from the 10 MMR^−^ strains (scale on right axis; adjusted to bring the wavelets together for comparison). (B and D) Plots of the wavelet coherences between the data from the tested strain and the data from the 10 MMR^−^ strains. (See the legend to Fig. 1 for further explanations of the bar charts and of the coherence plots.) Strains: panels A and B, Δ*nrdR* Δ*mutL* mutant strain PFM799; panels C and D, Δ*rep* Δ*mutL* mutant strain PFM677.

Loss of the NrdR repressor results in increased expression of ribonucleotide reductase (RNR) and thus in increased dNTP levels throughout the cell cycle (19, 20) (see Text S3 in the supplemental material for further discussion). We tested a Δ*nrdR* Δ*mutL* mutant strain and found that the BPS rate doubled, as expected when dNTP levels are high (Table 3). As shown Tables 1 and 2 (see also Fig. 5A and B), loss of NrdR did not change the basic BPS pattern, but the peak rate on each side was shifted about 200 kb away from the origin. This pattern was similar to that observed when cells were grown at low temperature (Fig. 4G).

**TABLE 3:** Experimental data

Strain	Description	No. of BPSs	No. of MA lines	Total no. of generations	BPSs per generation × 10^3^	95% CL^*d*^
Collective^*a*^	MMR^–^	30,061	334	264,958	113	2
PFM421	Δ*rnhA* Δ*mutL*	3,198	39	22,690	141	7
PFM669	Δ*mutL* (AB1157)	1,334	33	18,850	71	6
PFM430/431	*oriC^+^ oriZ^+^* Δ*mutL* (AB1157)	3,150	65	33,607	94	6
PFM426	Δ*oriC oriZ^+^* Δ*mutL (AB1157)*	3,544	45	25,176	141	9
PFM533/534	Δ*seqA* Δ*mutL*	1,855	44	20,927	89	7
PFM5m^*a*^	Δ*mutL* on minimal medium	1,435	48	28,197	51	3
PFM343m^*a*^	Δ*mutS* on minimal medium	1,608	46	26,280	61	4
PFM343VBs^*a*^	Δ*mutS* on supplemented minimal medium	2,476	39	23,017	108	5
PFM343dLB^*a*^	Δ*mutS* on diluted LB	3,546	40	22,495	158	8
PFM342LTM^*a*^	Δ*mutS* at low temperature	1,259	44	24,875	51	5
PFM799	Δ*nrdR* Δ*mutL*	5,272	42	23,581	224	12
PFM677	Δ*rep* Δ*mutL*	3,258	43	23,961	136	7
PFM256	Δ*tus* Δ*mutL*	3,672	28	32,967	111	10
PFM257	Δ*matP* Δ*mutL*	3,979	33	36,725	108	7
PFM422	Δ*recA* Δ*mutL*	6,797	40	39,713	171	11
PFM424	Δ*recA* Δ*mutS*	6,266	36	36,340	172	11
PFM456	Δ*recB* Δ*mutL*	3,321	46	20,598	161	9
PFM118^*a*^	Δ*umuDC* Δd*inB* Δ*mutL*	1,110	23	12,078	92	41
PFM120	*lexA3* Δ*sulA* Δ*mutL*	2,909	33	19,853	147	13
PFM259	Δ*hupB* Δ*mutL*	3,910	32	36,390	107	8
PFM258	Δ*hupA* Δ*mutL*	2,075	26	27,315	76	7
PFM317/318	Δ*fis* Δ*mutL*	7,616	35	37,731	202	14
PFM482	Δ*fis* Δ*mutS*	4,669	40	22,687	206	12
PFM741	Δ*hns* Δ*mutL*	3,367	46	25,551	132	5
PFM713	Δ*dps* Δ*mutL*	2,927	40	23,377	125	8
PFM163^*b*^	*mutD5*	13,625	26	3,481	3,915	344
PFM165/397/399^*b*^	*mutD5* Δ*mutL*	40,686	75	7,012	5,802	456
Collective^*a*^	Wild Type	1,933	341	2,015,066	0.96	0.10
Collective^*c*^	Bacillus subtilis *mutS*::Tn*10*	10,551	71	171,805	61	3

a Data are from reference 11.

b Data are from reference 10.

c Data represent combined results from four MA experiments performed with DK2140 (NCIB3610 *mutS*::Tn*10*), DK2141 (NCIB3610 *mutS*::Tn*10*), DK2142 (NCIB3610 *mutS*::Tn*10* Δ*comI*), and DK2143 (NCIB3610 *mutS*::Tn*10* Δ*comI*). There were no differences in mutation rates or spectra among these strains.

d 95% CL, 95% confidence limits to the BPSs per generation.

10.1128/mBio.01226-19.3 TEXT S3 Further discussion of the control of ribonuclease reductase by NrdR. Download Text S3, DOCX file, 0.01 MB. Copyright © 2019 Niccum et al. 2019 Niccum et al. This content is distributed under the terms of the Creative Commons Attribution 4.0 International license.

Replication fork progression is aided by the accessory replication helicase, Rep, which removes proteins bound to the DNA in front of the fork (21–24). Rep also aids in restarting replication forks after they stall or collapse (25, 26). As shown in Tables 1 and 2 (see also Fig. 5C and D), with the exception of a region close to the origin, loss of Rep disrupted the BPS rate pattern across the chromosome, suggesting that slowing or stalling the fork results in a random distribution of BPSs.

### Replication termination.

Most MA experiments showed that the BPS rate increased in the region where chromosomal replication terminates. Usually there were two unequal peaks, as shown in Fig. 1A, but in some experiments these peaks were better defined and of equal heights and, occasionally, there was just one peak. We do not know the source of this variation, but it may simply represent random noise.

**FIG 6:**
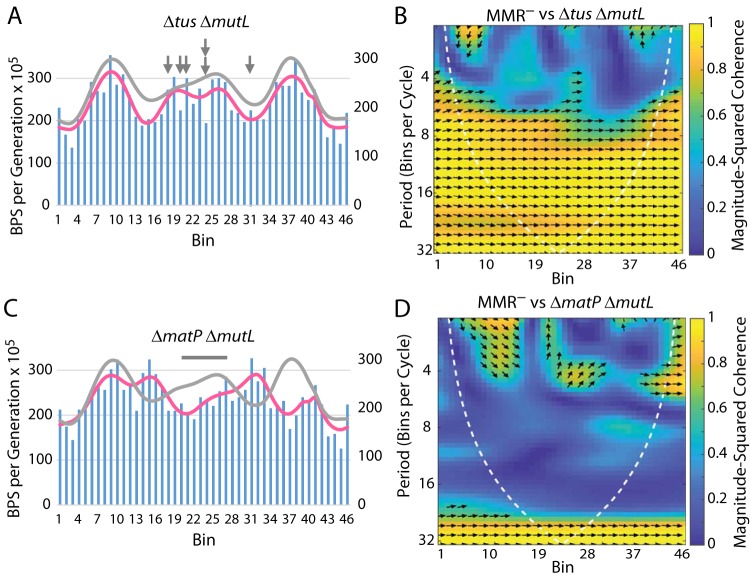
The effects on the BPS rate pattern of loss of the Tus antihelicase and MatP, the terminus organizing protein. (A and C) Bars represent the BPS rate in each bin for the tested strain; the pink lines represent the Daubechies wavelet transform of the binned data (scale on left axis). The gray lines represent the Daubechies wavelet transform of the binned data from the 10 MMR^−^ strains (scale on right axis; adjusted to bring the wavelets together for comparison). (B and D) Plots of the wavelet coherences between the data from the tested strain and the data from the 10 MMR^−^ strains. (See the legend to Fig. 1 for further explanations of the bar charts and of the coherence plots.) The arrows in panel A mark the major Ter sites where Tus binds. The bar in panel C shows the region in which MatP binds. Strains: panels A and B, Δ*tus* Δ*mutL* mutant strain PFM256; panels C and D, Δ*matP* Δ*mutL* mutant strain PFM257.

Replication terminates approximately 180° from the origin in a 1,200-kb region bounded by replication pause (Ter) sites; this region extends from bin 18 to bin 31 in our figures. The antihelicase Tus protein binds to the Ter sites and allows each replication fork to enter but not to exit, creating a replication fork “trap,” within which the two forks fuse and the chromosome dimer is resolved (27). To determine if the interaction of replication forks with Tus contributes to the increased mutation rate within this region, we performed an MA experiment with a Δ*tus* Δ*mutL* mutant strain. As shown in Tables 1 and 2 (see also Fig. 6A and B), loss of Tus had little effect on the BPS rate pattern.

The Ter MD extends from kb 1200 to kb 2200 (12), which is roughly from bin 20 to bin 28 in our figures. The structure of the Ter MD is maintained by the MatP protein, which binds to 23 *matS* sites within this region (28). As shown in Tables 1 and 2 (see also Fig. 6C and D), loss of MatP caused a severe disruption of the BPS pattern. The mutation rates in the Ter MD were depressed whereas new peaks appeared on either side of the Ter MD in the right and left MDs. Interestingly, the BPS pattern near the origin was maintained in the right replichore but not in the left replichore.

### Recombination.

Homologous recombination repairs and restarts the replisome after blockage (29), and the termination region is subject to hyperrecombination (30, 31). However, elimination of the major recombinase of E. coli, RecA, had only a modest effect on the BPS rate pattern in the Δ*mutL* or Δ*mutS* mutant strains (Tables 1 and 2; see also Fig. S1C and D in the supplemental material). BPS rates declined in bins 21 to 25, corresponding fairly well to the zone of hyperrecombination. However, this hyperrecombination is dependent on the RecA-RecBCD recombination pathway, and eliminating RecB did not reproduce the BPS pattern seen when RecA was absent (Tables 1 and 2; see also Fig. S1E and F in the supplemental material). Either our protocol is not sensitive enough to detect an effect of loss of RecB or another recombination pathway, e.g., RecFOR (32), is sufficient to maintain the BPS rate in the region.

### The SOS response.

In addition to its role in recombination, RecA is also a master regulator of the SOS response to DNA damage. MA experiments performed with a *lexA3* Δ*mutL* mutant strain that cannot induce the SOS response (33), and with a Δ*umuDC* Δ*dinB* Δ*mutL* mutant strain that is missing the error-prone DNA polymerases that are induced as part of the response (34), showed little effect on the BPS rate pattern (Tables 1 and 2; see also Fig. S2 in the supplemental material).

10.1128/mBio.01226-19.7 FIG S2 Lack of effect of loss of SOS functions on the BPS rate pattern. (A and C) Bars represent the mean BPS rate in each bin accumulated in the tested strain; the pink lines represent the Daubechies wavelet transform of the binned data (scale on left axis). The gray lines represent the Daubechies wavelet transform of the binned data from the 10 MMR^−^ strains (scale on right axis; adjusted to bring the wavelets together for comparison). (B and D) Plots of the wavelet coherences between the data from the tested strain and the data from the 10 MMR^−^ strains. (See the legend to Fig. S1 in the supplemental material for further explanation of bar charts and of the coherence plots.) The *lexA3* allele encodes a noninducible repressor of the SOS genes. Deletion of *sulA* prevents lethal filamentation after induction of the SOS response (not relevant to this study). The *dinB* and *umuDC* genes encode the error-prone DNA polymerases (Pol IV and Pol V) that are induced as part of the SOS response. Strains: panels A and B, *lexA3* Δ*sulA* Δ*mutL* mutant strain PFM120; panels C and D, Δ*umuDC* Δ*dinB* Δ*mutL* mutant strain PFM118. Download FIG S2, EPS file, 1.4 MB. Copyright © 2019 Niccum et al. 2019 Niccum et al. This content is distributed under the terms of the Creative Commons Attribution 4.0 International license.

### Nucleoid-associated proteins.

In a previous study (1), we found that the BPS pattern of a Δ*mutL* strain was correlated with the density of genes activated by the HU protein and repressed by the Fis protein. While both of these NAPs affect transcription, the general lack of correlation of the BPS rate with transcriptional levels (7) (see above) led us to hypothesize that mutation rates across the chromosome were correlated with areas of high DNA structure (1). To test this hypothesis, we performed MA experiments with MMR^–^ strains also defective for each of several NAPs.

**FIG 7:**
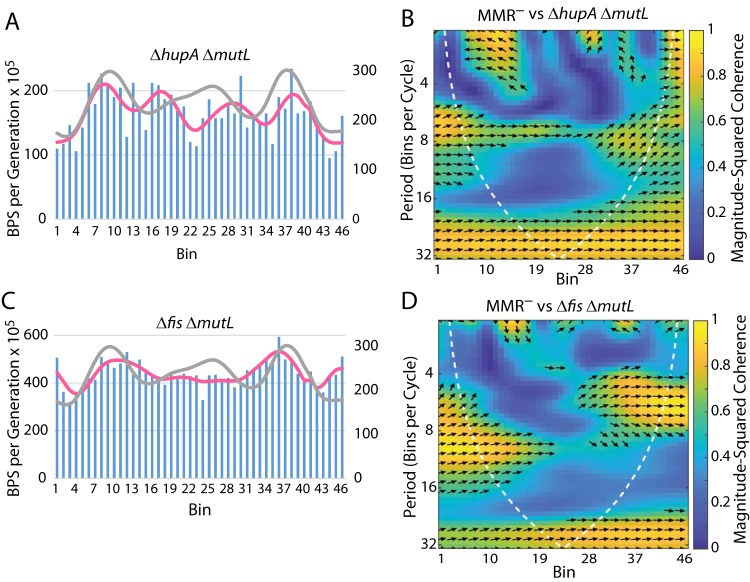
Role of HU and Fis in determining the BPS rate pattern. (A and C) Bars represent the BPS rate in each bin for the tested strain; the pink lines represent the Daubechies wavelet transform of the binned data (scale on left axis). The gray lines represent the Daubechies wavelet transform of the binned data from the 10 MMR^−^ strains (scale on right axis; adjusted to bring the wavelets together for comparison). (B and D) Plots of the wavelet coherences between the data from the tested strain and the data from the 10 MMR^−^ strains. (See the legend to Fig. 1 for further explanations of the bar charts and of the coherence plots.) Strains: panels A and B, Δ*hupA* Δ*mutL* mutant strain PFM258; panels C and D, Δ*fis* Δ*mutL* mutant strain PFM317/318.

HU exists as a dimer of its two subunits, HUα and HUβ, encoded by the paralogous genes *hupA* and *hupB*, respectively. While loss of both subunits confers a severe growth defect, loss of only one has little consequence during a normal growth cycle, suggesting they can substitute for each other. We performed MA experiments with both Δ*hupA* Δ*mutL* and Δ*hupB* Δ*mutL* mutant strains. Loss of *hupB* did not significantly affect the BPS pattern (Table 1). However, loss of *hupA* depressed BPS rates across the chromosome, particularly in the terminus region, while creating new peaks on either side of the Ter MD (Tables 1 and 2; see also Fig. 7A and B).

Sobetzko et al. (35) reported Fis binding sites clustered in the origin region; in contrast, both ChIP-chip (chromatin immunoprecipitation with microarray technology) and ChIP-Seq studies showed the density of Fis binding to be more or less constant across the chromosome (36, 37). In both Δ*fis* Δ*mutL* and Δ*fis* Δ*mutS* mutant strains (Tables 1 and 2) (Fig. 7B and C; see also Fig. S3A and B in the supplemental material), loss of Fis flattened the BPS rate pattern across the chromosome except around OriC.

10.1128/mBio.01226-19.8 FIG S3 The effect of loss of the NAPs Fis, HNS, and DPS on the BPS rate pattern. (A, C, and E) Bars represent the mean BPS rate in each bin accumulated in the tested strain; the pink lines represent the Daubechies wavelet transform of the binned data (scale on left axis). The gray lines represent the Daubechies wavelet transform of the binned data from the 10 MMR^−^ strains (scale on right axis; adjusted to bring the wavelets together for comparison). (B, D, and F) Plots of the wavelet coherences between the data from the tested strain and the data from the 10 MMR^−^ strains. (See the legend to Fig. S1 in the supplemental material for further explanation of bar charts and of the coherence plots.) Strains: panels A and B, Δ*fis* Δ*mutS* mutant strain PFM482; panels C and D, Δ*hns* Δ*mutL* mutant strain PFM741; panels E and F, Δ*dps* Δ*mutL* mutant strain PFM713. Download FIG S3, EPS file, 2.8 MB. Copyright © 2019 Niccum et al. 2019 Niccum et al. This content is distributed under the terms of the Creative Commons Attribution 4.0 International license.

HNS binds to DNA at high-affinity binding sites, spreads by oligomerization, and condenses the DNA into a few clusters per chromosome (38–40). However, as shown in Tables 1 and 2 (see also Fig. S3C and D in the supplemental material), loss of HNS had little effect on the BPS pattern.

The DPS protein accumulates in stationary-phase cells, condenses the nucleoid into a crystal-like state, and protects the DNA from oxidative and other damage (41). Despite this physical change, loss of DPS had little effect on the BPS rate pattern (Tables 1 and 2; see also Fig. S3E and F in the supplemental material). Of course, we do not know the degree to which cells in stationary phase contribute to the BPS rates under our experimental conditions.

### Proofreading.

**FIG 8:**
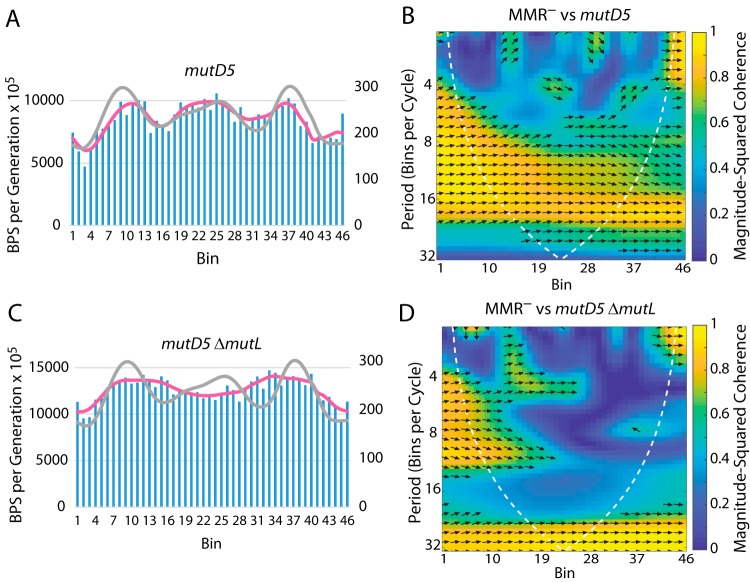
The role of proofreading in determining the BPS rate pattern. (A and B) Bars represent the BPS rate in each bin for the tested strain; the pink lines represent the Daubechies wavelet transform of the binned data (scale on left axis). The gray lines represent the Daubechies wavelet transform of the binned data from the 10 MMR^−^ strains (scale on right axis; adjusted to bring the wavelets together for comparison). (C and D) Plots of the wavelet coherences between the data from the tested strain and the data from the 10 MMR^−^ strains. (See the legend to Fig. 1 for further explanations of the bar charts and of the coherence plots.) The *mutD5* allele encodes an exonuclease-deficient proofreader. Strains: panels A and B, *mutD5* mutant strain PFM163; panels C and D, *mutD5* Δ*mutL* mutant strains PFM165/397/399.

The *mutD5* allele encodes an epsilon protein that is inactive for proofreading (42, 43). As shown in Tables 1 and 2 (see also Fig. 8A and B), when proofreading was inactive but MMR was active, the BPS rate pattern was less dramatic but basically the same as when only MMR was inactive. When both MMR and proofreading were inactive, which should reveal the mutations due to polymerase errors, the BPS pattern was nearly flat but still correlated with the MMR^−^ pattern except in the termination region (Tables 1 and 2; see also Fig. 8C and D).

### Wild type.

**FIG 9:**
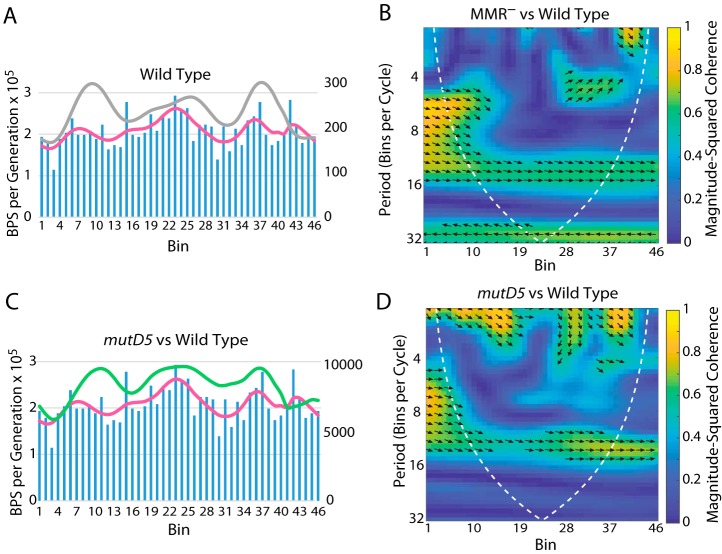
The BPS density pattern in the wild-type strain compared to the patterns of the MMR-defective strains and the *mutD5* mutant strain. (A and C) Bars represent the BPS rate in each bin for the wild-type strains, and the pink lines represent the Daubechies wavelet transform of the binned data (scale on left axis). The gray line in panel A represents the Daubechies wavelet transform of the binned data from the 10 MMR^−^ strains; the green line in panel C represents the Daubechies wavelet transform of the *mutD5* mutant strain (pink line in Fig. 8A) (scale on right axis; adjusted to bring the wavelets together for comparison). (B and D) Plots of the wavelet coherences between the data from the wild-type strains and the data from the 10 MMR^−^ strains (B) or the *mutD5* mutant strain (D). (See the legend to Fig. 1 for further explanations of the bar charts and of the coherence plots). Wild Type, eight strains with the wild-type mutational phenotypes (see the text).

The wave pattern of BPSs evident in our data, and in data from other bacteria (2–6), was obtained when MMR was inactive. Thus, a question arises. Does the pattern appear in wild-type strains? This question is difficult to answer because the low mutation rates of wild-type strains would necessitate enormous experiments in order to accumulate enough mutations to approach statistical confidence. In a recent study, we compared the mutation rates and spectra of E. coli strains defective in various DNA repair activities; among those, the results from seven strains were indistinguishable from those of the wild-type parent (44). By combining the data from these strains, we achieved 1,933 BPSs (11), enough to expect to see a wave pattern if it existed. As is evident in Tables 1 and 2 (see also Fig. 9), these BPSs did not create a pattern significantly correlated to the MMR^−^ pattern. However, the wild-type pattern is similar to both that seen in the MMR-defective strains and that seen in the *mutD5* mutant strain, particularly around the terminus.

### Bacillus subtilis.

**FIG 10:**
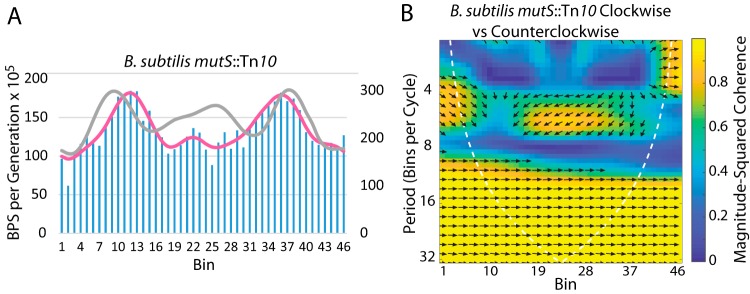
The BPS rate pattern of MMR-defective B. subtilis. (A) Bars represent the BPS rate in each bin for the B. subtilis MMR-defective strains; the pink line represents the Daubechies wavelet transform of the binned data (scale on left axis). The gray line represents the Daubechies wavelet transform of the binned data from the 10 E. coli MMR^−^ strains (scale on right axis; adjusted to bring the wavelets together for comparison). (B) Plot of the wavelet coherence between the binned BPS data from the B. subtilis strains taken in the clockwise and counterclockwise directions around the chromosome. (See the legend to Fig. 1 for further explanations of the bar charts and of the coherence plots). Strains: four B. subtilis *mutS*::Tn*10* strains with the same mutational phenotype (Table 3) (see also Table S1 in the supplemental material).

To date, BPS patterns that are symmetrical across the chromosome have been found only in MMR^−^ derivatives of Gram-negative bacteria (1–6). Here we add the Gram-positive bacterium Bacillus subtilis to this list. As shown in Table 2 (see also Fig. 10), the BPS rates across the chromosome in B. subtilis *mutS*::Tn*10* mutant strains fell into a wave-like pattern that was symmetrical about the origin. Although similar in shape, the pattern was significantly different from that of E. coli (Table 1). However, as in E. coli, the BPS rate appeared to increase in the terminus region, which in B. subtilis is not 180° from the origin and corresponds to bins 20 to 24 (Fig. 10).

10.1128/mBio.01226-19.9 TABLE S1 Bacterial strains used in this study. Download Table S1, DOCX file, 0.02 MB. Copyright © 2019 Niccum et al. 2019 Niccum et al. This content is distributed under the terms of the Creative Commons Attribution 4.0 International license.

## DISCUSSION

In this study, we examined the role that a number of factors play in establishing and maintaining the symmetrical wave-like BPS rate pattern across the E. coli chromosome. These factors were as follows: transcription, replication initiation and progression, replication termination, recombination, the SOS response to DNA damage, nucleoid-associated proteins, and error correction by MMR and proofreading. We found that transcription and the SOS response had little effect and that the effect of recombination was modest and confined to the terminal region. Here we discuss only the more significant factors.

### Replication initiation.

Providing additional replication origins, either by eliminating RNase H1 or by inserting an ectopic *oriC* (*oriZ*) gene (Tables 1 and 2), did not disrupt the wave pattern. However, when *oriZ* was the only origin of replication, the region of depressed BPS rates that surrounds *oriC* was reestablished about the new origin (Fig. 3). Only 5.1 kb of DNA containing *oriC* was relocated (15), whereas the region of reduced BPS rate is about 200 kb; thus, the BPS rate is not determined just by the DNA sequence surrounding *oriC*. We hypothesize that the process of replication initiation protects the DNA from damage and/or that newly established replication forks have a low error rate. In support of the second hypothesis, replication forks have been found to frequently pause in a 200-kb zone on either side of the origin (45), which could allow more-efficient error correction.

The BPS mutation rate increased for about 1,000 kb (10 bins) on either side of OriZ such that the pattern resembled that of the same region normally about OriC. An ectopic origin was found to change the MD structure of the chromosome over similar lengths, although the mechanism of this remodeling is unknown (46). In the presence of OriZ, however, the BPS pattern across the rest of the chromosome remained symmetrical about the absent OriC (Fig. 3). Thus, the nature of the BPS rate pattern appears to be due to at least two factors: one factor affecting only the origin and another factor or factors determining the pattern elsewhere on the chromosome.

### Replication progression.

As mentioned above, Dillon et al. (2) identified the timing of replication as a significant determinant of the BPS rate pattern. They suggested that the pattern reflects variations in dNTP levels that occur as origins fire during rapid growth. At replication initiation, a burst of RNR activity increases the dNTP levels, but as the fork progresses, dNTP levels fall, becoming limiting for replication. During rapid growth, initiation of each additional fork again increases dNTP levels, but these fall rapidly as the forks progress. These fluctuations in dNTP levels should result in fluctuations in replication accuracy, since accuracy is inversely related to dNTP levels (2). High dNTP levels increase the probability of misincorporation, whereas limiting levels of dNTPs slow polymerization, allowing error correction by proofreader and MMR to prevail (47).

Our results provide partial support for this hypothesis. Dysregulation of RNR in the Δ*nrdR* mutant strain shifted the BPS rate pattern away from the origin (Fig. 5A and B), which is expected if the abnormal elevation of dNTP levels delayed the point at which they became limiting. Slowing cell growth by lowering the temperature caused a similar shift in the pattern, also expected if the initiation of fewer forks kept dNTP levels elevated (Fig. 4G and H). However, our results also indicate that growth on rich medium, rather than the growth rate *per se*, is a significant determinant of the BPS pattern (Fig. 4A to F), possibly because of effects on the expression and DNA binding of HU and Fis (see below).

Loss of the accessory replication helicase (Rep) resulted in a nearly complete loss of the BPS pattern (Fig. 5C and D), suggesting that frequent and, presumably, random replication stalling causes mutation rates to become chaotic. An alternative, intriguing possibility is that Rep is responsible for modulating the interactions of the replication fork with the NAPs, whose binding determines the BPS rate pattern across much of the chromosome.

### Replication termination.

After declining to a local minimum about 3/5 of the distance along each replichore, BPS rates rise in the terminus region (Fig. 1A). Eliminating MatP, the protein that maintains the structure of the Ter MD, reduced these rates (Fig. 6C and D), suggesting that replication of highly structured DNA is error prone. Interestingly, BPS rates increased in the right and left MDs in Δ*matP* mutant cells, suggesting that these adjacent regions of DNA gained structure in the absence of MatP. The mechanism by which MatP structures the Ter MD is not entirely clear (48, 49), but it probably involves supercoils, which, when unconstrained, could migrate into adjacent areas.

### Nucleoid-associated proteins.

From our previous results, we predicted that the NAPs HU and Fis, but not HNS, have a role in establishing or maintaining the BPS rate pattern (1). The results presented here (Tables 1 and 2) (Fig. 7) confirmed those predictions and additionally show that DPS had no effect on the BPS rate pattern. Because the NAPs affect gene expression in various ways, we cannot conclude that DNA binding by the NAPs themselves is responsible for the mutational pattern. And, indeed, we found no significant correlations between the BPS pattern and published binding sites of the NAPs, although the locations of the binding sites themselves differ widely among published results (see, e.g., references 37, 50, and 51). Nonetheless, we favor the hypothesis that structuring of the DNA mediated by the NAPs, directly or indirectly, contributes to the BPS rate pattern. Specifically, we hypothesize that DNA replication becomes inaccurate in regions of high superhelical density.

The local effect of HU binding is to bend the DNA, but dimer-dimer interactions produce higher-order HU-DNA complexes that can constrain negative supercoils (52, 53). (See Text S4 in the supplemental material for further discussion of the HU subunits.) While loss of HUβ had little effect on the BPS rate pattern (Table 1), loss of HUα reduced the overall BPS rate by 33% (Table 3) and also changed the pattern (Fig. 7A and B). BPS rates were low in the terminus region but were elevated in the right and left MDs, results which were very similar to the pattern seen in the Δ*matP* Δ*mutL* mutant strain (Fig. 6C and D).

Fis is a major transcriptional regulator, either activating or repressing, directly or indirectly, nearly a thousand genes (36). Fis acts as a classic transcriptional regulator at some promoters but affects gene expression by altering the DNA superhelical density at other promoters (54). The loss of Fis nearly doubled the overall BPS rate (Table 3) but flattened the wave pattern outside the origin region (Fig. 7C and D).

In addition to local effects, recently developed chromosome conformation capture techniques have revealed that NAPs participate in the overall structuring of the chromosome (49). HU cooperates with the MukBEF condensin to promote long-range chromosomal interactions, while Fis acts similarly, although without the cooperation of MukBEF. Within the Ter domain, MatP restricts MukBEF activity, creating a distinct domain with short-range interactions, but HU and Fis partially counteract this restriction. Although these studies did not address how long-range DNA interactions are achieved, a likely candidate for the factor responsible is DNA supercoiling. Supercoil diffusion brings distant loci together as the two DNA strands writhe about each other (55). While not all of our results can be explained by those newly determined data, the effects of NAPs and MatP on the BPS rate pattern can be explained if replication accuracy is deceased in regions of high superhelical density.

### Error correction.

Assuming that the BPSs recovered from the *mutD5* Δ*mutL* mutant strain were due to intrinsic errors made by DNA polymerase, we conclude that the polymerase is accurate close to the origin and then becomes increasingly less accurate as replication proceeds to about 1/3 of the replichore, at which point accuracy increases again (Fig. 8C and D). The patterns of BPSs in the MMR-defective strains reveal proofreader biases, whereas the patterns of BPSs in the *mutD5* mutant strain reveal MMR biases. Since the BPS patterns in the *mutD5* mutant strain and the MMR-defective strains are similar (Fig. 8A and B), error correction by MMR and error correction by proofreader activity apparently have the same biases, although MMR is much less powerful. Thus, error correction by each is effective close to the origin, declines for about 1/3 of the replichore, and increases in the right and left MDs but declines again in the terminus region (Fig. 1; see also Fig. 8A and B). This pattern probably reflects the combined effects of dNTP pool fluctuations and NAP-induced DNA structure, both of which would affect the error correction activities of proofreader and MMR.

Given the considerations described above, we would expect the BPS rate pattern in the wild-type strain to mimic that of the MMR-defective strains. However, the nearly 2,000 BPSs accumulated in phenotypically wild-type strains appear to be randomly distributed across the chromosome (Fig. 9). One explanation for this discrepancy is that when both proofreading and MMR are active, the mutation rate is reduced to the point that other, weaker DNA repair and mutagenic activities obscure the underlying pattern. Although the data are not significantly correlated, the wild-type BPS pattern is similar to both the MMR^–^ pattern and the *mutD5* mutant pattern, particularly around the terminus, supporting this hypothesis. Alternatively, although the eight strains that we combined to produce 2,000 BPSs have the same mutation rates and spectra, they may have different wave patterns that tend to negate each other and obscure the underlying pattern.

## MATERIALS AND METHODS

### Bacterial strains and media.

The strains used in this study are listed in Table S1 in the supplemental material, and the methods of their construction are described in Text S5 in the supplemental material. The oligonucleotides used in this study are listed in Table S2 in the supplemental material. Standard media and antibiotics were used (see Text S5 in the supplemental material).

10.1128/mBio.01226-19.4 TEXT S4 Further discussion of the HU subunits. Download Text S4, DOCX file, 0.01 MB. Copyright © 2019 Niccum et al. 2019 Niccum et al. This content is distributed under the terms of the Creative Commons Attribution 4.0 International license.

10.1128/mBio.01226-19.5 TEXT S5 Supplemental material and methods. Download Text S5, DOCX file, 0.02 MB. Copyright © 2019 Niccum et al. 2019 Niccum et al. This content is distributed under the terms of the Creative Commons Attribution 4.0 International license.

10.1128/mBio.01226-19.10 TABLE S2 Oligonucleotides used in this study. Download Table S2, DOCX file, 0.01 MB. Copyright © 2019 Niccum et al. 2019 Niccum et al. This content is distributed under the terms of the Creative Commons Attribution 4.0 International license.

### Mutation accumulation experiments.

The MA procedure has been described (7, 11, 44). Details are given in the Text S5 in the supplemental material.

### Genomic DNA preparation, library construction, sequencing, and sequence analysis.

The methods used for genomic DNA preparation, library construction, sequencing, and sequence analysis have been described previously (7, 11, 44). Details are given in the Text S5 in the supplemental material.

### RNA sequencing.

Strain PFM144, which is PFM2 Δ*mutL* (11), was grown in LB, and aliquots, each containing the same number of cells, were collected during the lag (optical density [OD] = 0.022), log (OD = 0.3), and stationary (OD = 1.5) phases. Total RNA was extracted, DNA and rRNA were removed, and libraries were constructed and sequenced as detailed in Text S5 in the supplemental material. Three biological triplicates were prepared for each growth phase.

The numbers of RNA-Seq reads for each condition were normalized to the number of reads mapped to the *holD* gene, which was shown by real-time PCR (rtPCR) to be expressed at the same level in all phases of growth. The means of the normalized RNA reads from the triplicates were then binned into the same bins used for the mutational analysis. A fourth-order Daubechies wavelet transform was performed on the binned RNA-Seq reads as described for the mutational data (1).

### Statistical analysis.

To obtain the BPS density patterns, the numbers of BPSs were binned into 46 bins, each ∼100 kb long, as described previously (1). A fourth-order Daubechies wavelet transform was performed on the binned mutation data as described previously (1). For presentation in the figures, these results were converted into rate values by dividing the number of BPSs by the appropriate number of generations. Pearson’s product-moment correlation coefficient, ρ, was used to evaluate the correlations between the binned BPS data (Tables 1 and 2). Spearman’s nonparametric correlation coefficient was also computed for a few data sets but gave similar results. To account for multiple comparisons, *P* values were adjusted using the Benjamini-Hochberg method (56) with the false-discovery rate set at 25%, implemented with the MatLab R2018a “mafdr” command. Because comparisons performed using data from the same strain are not independent, this adjustment was made separately for each column in Tables 1 and 2.

To further compare the BPS density patterns between two data sets, wavelet coherence was calculated and plotted using the MatLab R2018a “wcoherence” command. While the Daubechies wavelet provides a good visual representation of the binned data, it is not continuous and thus not easily adapted for wavelet coherence analysis. The MatLab program first converts the binned data to Morlet wavelets and then computes the coherence between two of these wavelets. We chose to analyze the data with wavelet coherence because it gives a measure of the correlation between the signals (displayed as colors in the figures) (57, 58). In addition, the MatLab wavelet coherence plot indicates, as a dashed curve, the “cone of influence” within which results are free of artifactual edge effects (57). The relative levels of phase lag between the two signals are indicated by small arrows: arrows pointing right indicate in phase, arrows pointing left indicate 180° out of phase, and arrows pointing in other directions indicate the various degrees between. Because the MatLab program assumes a frequency-time series, the *x* axis of the plot represents cycles/sample and the *y* axis represents time; we converted these to bins/cycle (on an inverted scale) and bins, respectively.

### Data availability.

The sequences and SNPs reported in this paper were deposited with the National Center for Biotechnology Information Sequence Read Archive (https://trace.ncbi.nlm.nih.gov/Traces/sra/) (accession no. PRJNA168337 for E. coli and accession no. PRJNA542344 for B. subtilis) and in the IUScholarWorks Repository (http://hdl.handle.net/2022/23028).
