# Supplementary material for: The Symmetrical Wave Pattern of Base-Pair Substitution Rates across the Escherichia coli Chromosome Has Multiple Causes
Source: mBio. 2019 Jul 2;10(4):e01226-19. doi: 10.1128/mBio.01226-19 (PMC6606806; doi:10.1128/mBio.01226-19)
Supplement: TEXT S1 [file mBio.01226-19-s0001.docx]

**Text S1**

Because ribosomal operons are homologous, we could not call SNPs in these genes. The RNA reads from the genes in ribosomal operons were also removed from the RNA-Seq data, but their positions have been indicated in Fig. 2A. Bin 35, which includes the ribosomal *rrnG* operon, had a large number of RNA reads even in the absence of reads from the *rrnG* genes. This high level of expression is almost exclusively due to the *ssrA* gene that encodes transfer-messenger RNA (1) and is highly expressed under all three conditions (the RNA-Seq data will be further analyzed in a subsequent paper). The BPS rate pattern of several of the strains reported in the main text reach a minimum in this general area, but it is as often at bin 33 as at bin 35. Given that the bin size is 100 Kb, it seems unlikely that the high level of transcription level of *ssrA*, located in the middle of bin 35, is causing these patterns.

Mutation rates have been reported to be both increased (2) and decreased (3) by high levels of transcription. In a previous paper (4) we reported that highly expressed genes had normal mutation rates, a finding that was confirmed in a recent study using deep sequencing to detect spontaneous mutations in *E. coli* (5).

**References**

1. Withey JH, Friedman DI. 2003. A salvage pathway for protein structures: tmRNA and trans-translation. Annu Rev Microbiol 57:101-23.

2. Jinks-Robertson S, Bhagwat AS. 2014. Transcription-associated mutagenesis. Annu Rev Genet 48:341-59.

3. Rasouly A, Pani B, Nudler E. 2017. A magic spot in genome maintenance. Trends Genet 33:58-67.

4. Lee H, Popodi E, Tang H, Foster PL. 2012. Rate and molecular spectrum of spontaneous mutations in the bacterium *Escherichia coli* as determined by whole-genome sequencing. Proc Natl Acad Sci USA 109:E2774-E2783.

5. Zhang X, Zhang X, Zhang X, Liao Y, Song L, Zhang Q, Li P, Tian J, Shao Y, Ai-Dherasi AM, Li Y, Liu R, Chen T, Deng X, Zhang Y, Lv D, Zhao J, Chen J, Li Z. 2018. Spatial vulnerabilities of the *Escherichia coli* genome to spontaneous mutations revealed with improved duplex sequencing. Genetics 210:547-558.
