# Supplementary material for: The Symmetrical Wave Pattern of Base-Pair Substitution Rates across the Escherichia coli Chromosome Has Multiple Causes
Source: mBio. 2019 Jul 2;10(4):e01226-19. doi: 10.1128/mBio.01226-19 (PMC6606806; doi:10.1128/mBio.01226-19)
Supplement: TEXT S2 [file mBio.01226-19-s0002.docx]

**Text S2**

In *E. coli* the adenines in GATC sites are methylated by the Dam methylase. The SeqA protein binds to hemimethylated GATC sites, many of which are clustered around OriC, and, by so doing, SeqA hinders origin firing. In the absence of SeqA, unregulated initiation presumably results in over-replication, at least when cells are rapidly growing in rich medium (1). Downstream events, such as replication fork collapse, add to the phenotypes of *seqA* mutant cells (2).

By binding to hemimethylated DNA, SeqA forms complexes behind the replication fork as it progresses around the chromosome (1). In addition, SeqA binds to areas of the chromosome with closely spaced GATC sites, as well as to particular genes regulated by GATC methylation (3). In the absence of SeqA the superhelicity of the chromosome increases, the nucleoid condenses (4), and transcription is altered (5).

As shown in Tables 1 and 2 and Fig.S1 A,B in the supplementary material, loss of SeqA somewhat amplified the BPS density pattern of the right replichore, but the pattern was still highly correlated to that of right replichore of the MMR-defective strains. However, the pattern in the left replichore was disrupted in the Δ*seqA* Δ*mutL* mutant strain. Based on chromatin immunoprecipitation analysis, this area of the left replichore is not targeted by SeqA to a greater extent than the same area of the right replichore (3, 6), suggesting that the disruption of the mutational pattern is not due to loss of binding by SeqA. As shown in Fig. 1A, bins 44, 38, 37, and 35 contain a number of highly expressed genes; in addition, bins 42 and 35 contain highly transcribed ribosomal RNA genes. Thus, we hypothesize that loss of SeqA makes the replication machinery particularly susceptible to interference by transcription, disrupting the mutational pattern. If this hypothesis is correct, the interference apparently makes replication more accurate, perhaps by slowing the speed of DNA polymerase.

**References**

1. Waldminghaus T, Skarstad K. 2009. The *Escherichia coli* SeqA protein. Plasmid 61:141-50.

2. Sutera VA, Jr., Lovett ST. 2006. The role of replication initiation control in promoting survival of replication fork damage. MolMicrobiol 60:229-239.

3. Sanchez-Romero MA, Busby SJ, Dyer NP, Ott S, Millard AD, Grainger DC. 2010. Dynamic distribution of SeqA protein across the chromosome of *Escherichia coli* K-12. mBio 1:e00012-10.

4. Weitao T, Nordstrom K, Dasgupta S. 2000. *Escherichia coli* cell cycle control genes affect chromosome superhelicity. EMBO Rep 1:494-9.

5. Lobner-Olesen A, Marinus MG, Hansen FG. 2003. Role of SeqA and Dam in *Escherichia coli* gene expression: a global/microarray analysis. Proc Natl Acad Sci U S A 100:4672-7.

6. Waldminghaus T, Skarstad K. 2010. ChIP on Chip: surprising results are often artifacts. BMC Genomics 11:414.
