# Supplementary material for: The Symmetrical Wave Pattern of Base-Pair Substitution Rates across the Escherichia coli Chromosome Has Multiple Causes
Source: mBio. 2019 Jul 2;10(4):e01226-19. doi: 10.1128/mBio.01226-19 (PMC6606806; doi:10.1128/mBio.01226-19)
Supplement: TEXT S3 [file mBio.01226-19-s0003.docx]

**Text S3**

In *E. coli* growing aerobically dNTP levels are regulated by a Class Ia ribonucleotide reductase (RNR) encoded by the *nrdA* and *nrdB* genes. The *nrdAB* operon is briefly upregulated when DNA replication initiates, resulting in a burst of RNR activity that then rapidly returns to basal levels (1). Although most of this regulation appears to be due to DnaA interactions (2), the NrdR repressor, which regulates a poorly expressed Class Ib RNR, also regulates *nrdAB* transcription; loss of NrdR results in increased expression of RNR throughout the cell cycle (3, 4). Increased RNR should result in increased dNTP levels, and, indeed, the Δ*nrdR* Δ*mutL* mutant strain had twice the mutation rate as the MMR– strains, as expected when dNTP levels are high (Table S1 in the supplementary material).

**References**

1. Sun L, Fuchs JA. 1992. *Escherichia coli* ribonucleotide reductase expression is cell cycle regulated. Mol Biol Cell 3:1095-105.

2. Gon S, Camara JE, Klungsoyr HK, Crooke E, Skarstad K, Beckwith J. 2006. A novel regulatory mechanism couples deoxyribonucleotide synthesis and DNA replication in *Escherichia coli*. EMBO J 25:1137-47.

3. Torrents E, Grinberg I, Gorovitz-Harris B, Lundstrom H, Borovok I, Aharonowitz Y, Sjoberg BM, Cohen G. 2007. NrdR controls differential expression of the *Escherichia coli* ribonucleotide reductase genes. J Bacteriol 189:5012-21.

4. Zhu M, Dai X, Guo W, Ge Z, Yang M, Wang H, Wang YP. 2017. Manipulating the bacterial cell cycle and cell size by titrating the expression of ribonucleotide reductase. mBio 8:e01741-17.
