# Supplementary material for: The Symmetrical Wave Pattern of Base-Pair Substitution Rates across the Escherichia coli Chromosome Has Multiple Causes
Source: mBio. 2019 Jul 2;10(4):e01226-19. doi: 10.1128/mBio.01226-19 (PMC6606806; doi:10.1128/mBio.01226-19)
Supplement: TABLE S1 [file mBio.01226-19-st001.docx]

| **Table S1. Bacterial strains used in this study** | | | | | |
| --- | --- | --- | --- | --- | --- |
| Strain | Relevant genotype | Donor | Recipient | Target gene | Reference |
| PFM2 | MG1655 *rph^+^* |  |  |  | (1) |
| PFM5 | Δ*mutL* |  |  |  | (1) |
| WX320 | Δ*oriC oriZ^+^* (AB1157) |  |  |  | (2) |
| WX340 | *oriC^+^ oriZ^+^* (AB1157) |  |  |  | (2) |
| PF11 | AB1157 |  |  |  | Lab strain |
| PFM118 | Δ*umuDC* Δ*dinB* Δ*mutL* |  |  |  | (3) |
| PFM120 | *lexA3* Δ*sulA* Δ*mutL* |  |  |  | This Study |
| PFM163 | *mutD5* |  |  |  | (4) |
| PFM165/397/399 | *mutD5* Δ*mutL* |  |  |  | (4) |
| PFM244 | Δ*mutL, scarless* |  |  |  | (3) |
| PFM256 | Δ*tus* Δ*mutL* | JW1602 | PFM244 | Δ*tus::*Kn*^R^* | This Study |
| PFM257 | Δ*matP* Δ*mutL* | JW0939 | PFM244 | Δ*matP::*Kn^R^ | This Study |
| PFM258 | Δ*hupA* Δ*mutL* | JW3964 | PFM244 | Δ*hupA::*Kn^R^ | This Study |
| PFM259 | Δ*hupB* Δ*mutL* | JW0430 | PFM244 | Δ*hupB:*:Kn^R^ | This Study |
| PFM317/ 318 | Δ*fis* Δ*mutL* | JW3229 | PFM244 | Δ*fis::*Kn^R^ | This Study |
| PFM342/343 | Δ*mutS, scarless* |  |  |  | (3) |
| PFM421 | Δ*rnhA* Δ*mutL* | JW0204 | PFM244 | Δ*rnhA:*:Kn^R^ | This Study |
| PFM422 | Δ*recA* Δ*mutL* | JW2669 | PFM244 | Δ*recA::*Kn^R^ | This Study |
| PFM424 | Δ*recA* Δ*mutS* | JW2669 | PFM342 | Δ*recA::Kn^R^* | This Study |
| PFM426 | Δ*oriC oriZ*^+^ Δ*mutL* (AB1157) | JW4128 | WX320 | Δ*mutL::*Kn^R^ | This Study |
| PFM430/ 431 | *oriC*^+^ *oriZ*^+^ Δ*mutL* (AB1157) | JW4128 | WX340 | *ΔmutL::*Kn^R^ | This Study |
| PFM456 | Δ*recB* Δ*mutL* | JW2788 | PFM244 | Δ*recB::*Kn^R^ | This Study |
| PFM482 | Δ*fis* Δ*mutS* | JW3229 | PFM343 | Δ*fis::*Kn^R^ | This Study |
| PFM533/ 534 | Δ*seqA* Δ*mutL* | JW0674 | PFM244 | Δ*seqA::*Kn^R^ | This Study |
| PFM661 | Δ*hupA* ΔrecA Δ*mutL* | JW2669 | PFM258 | Δ*recA::Kn^R^* | This Study |
| PFM669 | Δ*mutL* (AB1157) | JW4128 | PF11 | Δ*mutL::*Kn^R^ | This Study |
| PFM677 | Δ*rep* Δ*mutL* | JW5604 | PFM244 | Δ*rep::*Kn^R^ | This Study |
| PFM713 | Δ*dps* Δ*mutL* | JW0797 | PFM244 | Δ*dps::*Kn^R^ | This Study |
| PFM741 | Δ*hns* Δ*mutL* | JW1225-2 | PFM244 | Δ*hns::*Kn^R^ | This Study |
| PFM799 | Δ*nrdR ΔmutL* | JW0403 | PFM244 | Δ*nrdR:*:Kn^R^ | This Study |
| DK2140/2141/ 2142/2143 | *Bacillus subtilis mutS::*Tn*10* |  |  |  | M. Konkol & D. Kearns, personal communication |

In all strains the Kn^R^ element was removed by FLP recombination (5).

Scarless: Scarless gene deletion using a cat‐I‐SceI cassette (6).

**References**

1. Lee H, Popodi E, Tang H, Foster PL. 2012. Rate and molecular spectrum of spontaneous mutations in the bacterium *Escherichia coli* as determined by whole-genome sequencing. Proc Natl Acad Sci USA 109:E2774-E2783.

2. Wang X, Lesterlin C, Reyes-Lamothe R, Ball G, Sherratt DJ. 2011. Replication and segregation of an Escherichia coli chromosome with two replication origins. Proc Natl Acad Sci U S A 108:E243-50.

3. Foster PL, Niccum BA, Popodi E, Townes JP, Lee H, MohammedIsmail W, Tang H. 2018. Determinants of base-pair substitution patterns revealed by whole-genome sequencing of DNA mismatch repair defective *Escherichia coli*. Genetics 209:1029-1042.

4. Niccum BA, Lee H, MohammedIsmail W, Tang H, Foster PL. 2018. The spectrum of replication errors in the absence of error correction assayed across the whole genome of *Escherichia coli*. Genetics 209:1043-1054.

5. Datsenko KA, Wanner BL. 2000. One-step inactivation of chromosomal genes in *Escherichia coli* K-12 using PCR products. Proc Natl Acad Sci USA 97:6640-6645.

6. Blank K, Hensel M, Gerlach RG. 2011. Rapid and highly efficient method for scarless mutagenesis within the *Salmonella enterica* chromosome. PLoS One 6:e15763.
