# Supplementary material for: The Symmetrical Wave Pattern of Base-Pair Substitution Rates across the Escherichia coli Chromosome Has Multiple Causes
Source: mBio. 2019 Jul 2;10(4):e01226-19. doi: 10.1128/mBio.01226-19 (PMC6606806; doi:10.1128/mBio.01226-19)
Supplement: TEXT S4 [file mBio.01226-19-s0004.docx]

**Text S4**

HUαβ is the dominant form over most of the cell cycle, but significant amounts of HUα_2_ are found during lag phase and early exponential phase, and HUβ_2_ is prominent in stationary phase (1). Chromatin immunoprecipitation sequencing (ChIP-Seq) results revealed that HU binds non-specifically to the chromosome and the DNA binding patterns of the three dimers appear to be identical (2).

**References**

1. Claret L, Rouviere-Yaniv J. 1997. Variation in HU composition during growth of *Escherichia coli*: the heterodimer is required for long term survival. J Mol Biol 273:93-104.

2. Prieto AI, Kahramanoglou C, Ali RM, Fraser GM, Seshasayee AS, Luscombe NM. 2012. Genomic analysis of DNA binding and gene regulation by homologous nucleoid-associated proteins IHF and HU in *Escherichia coli* K12. Nucleic Acids Res 40:3524-3537.
