# Supplementary material for: The Symmetrical Wave Pattern of Base-Pair Substitution Rates across the Escherichia coli Chromosome Has Multiple Causes
Source: mBio. 2019 Jul 2;10(4):e01226-19. doi: 10.1128/mBio.01226-19 (PMC6606806; doi:10.1128/mBio.01226-19)
Supplement: TEXT S5 [file mBio.01226-19-s0005.docx]

**Text S5**

**Supplemental Material and Methods**

**Bacterial strains and media.** The strains used in this study are listed in Table S2 in the supplemental material. All *E. coli* strains were derived from PFM2 (1) or AB1157 (2). The *oriC+ oriZ+* and Δ*oriC oriZ+* strains were a gift from Rodrigo Reyes-Lamothe (McGill University); Roel Schaaper (NIEHS) generously supplied the *mutD5* allele. Deletion mutations originated in the Keio collection (3) and were moved by P1 phage transduction (4); the Kn^r^ element was removed by using FLP recombination (5). The deletions were confirmed by PCR analysis using the oligonucleotides listed in Table S3 of the supplemental material. The *B. subtilis* strains were derived from the undomesticated ancestral strain NCIB3610 and were a gift from M.A. Konkol and D.B. Kearns (Indiana University).

Rich medium was Miller Luria Broth (LB) (Difco; BD); minimal medium was Vogel-Bonner minimal medium (VB min) with 0.2% glucose (6). When required, antibiotic concentrations were: carbenicillin (Carb), 100 μg/ml; kanamycin (Kn), 50 μg/ml; nalidixic acid (Nal), 40 μg/ml; chloramphenicol (Cam), 30 μg/ml; and, rifampicin (Rif), 100 μg/ml. Half of these concentrations were used in minimal medium.

**Estimation of mutation rates from fluctuation assays.** Mutation rates were estimated as described (7), using mutation to Nal^R^ or Rif^R^. The Ma-Sandri-Sarkar maximum likelihood method was used to calculate the mutation rates by using the FALCOR web tool found at www.mitochondria.org/protocols/FALCOR.html (8). These preliminary rates were used to determine the number of generations required for a MA experiment; final mutation rates given in Table S1 in the supplemental material were calculated from the results of the MA experiments.

**Mutation accumulation experiments.** The MA procedure has been described (1, 9, 10). The MA lines originated from single colonies isolated from a founder colony, obtained by streaking from a freezer stock onto agar plates of the medium to be used in the MA experiment. After incubation overnight at the experimental temperature, one well isolated colony was excised from the agar plate, soaked for 30 minutes in 0.85% NACL + 0.01% gelatin, and then vortexed for 60 seconds. Appropriate dilutions for obtaining well-isolated colonies were then plated onto the appropriate agar plates at the appropriate temperature to start MA lines. Plates were incubated at 37°C for most experiments, or at 28^o^C for the experiment at low temperature. Each MA line was periodically streaked for a single colony: on LB and supplemented VB min agar plates at 37^o^C, this was done daily; on VB min and diluted LB agar plates at 37^o^C, and on LB plates at 28^o^C, this was done every 48 hours. The number of passes required was determined by the preliminary mutation rates obtained from fluctuation assays. The parameters of the MA experiment, including the number of lines used for each strain and the total number of generations per experiment, are given in Table S1 in the supplemental material.

**Estimation of generations.** The method to estimate that number of generations undergone in each MA experiment is described (1, 9 ). The diameter of the single colonies streaked was recorded daily, and then the number of cells in colonies of different diameters was determined for each experiment, as described (1). The daily colony diameters were converted to generations and the results summed.

**Genomic DNA preparation, library construction, and sequencing.** Genomic DNA was isolated using PureLink Genomic DNA purification kit (Invitrogen) from overnight cultures inoculated from the freezer stocks made after the last passage of each MA line. DNA concentration was measured on an Epoch Microplate Spectrophotometer (BioTek Instruments, Inc.). The identity of the lines was confirmed before library construction by verifying the presence of the expected gene deletions in the gDNA with diagnostic PCR using the oligonucleotides in Table S3 in the supplemental material. Libraries were made by the Indiana University Center for Genomics and Bioinformatics and were sequenced using the Illumina HiSeq 2500 platform at the University of New Hampshire Hubbart Center for Genome Studies or the Illumina NextSeq platform at the Indiana University Center for Genomics and Bioinformatics.

**Sequence analysis.** SNP calling is described (1). The source code used, entitled MA-pipeline, is available at https://github.com/COL-IU/MA-pipeline. The reference genome used for *E. coli* was NCBI reference sequence NC_000913.2, and for *B. subtilis* was GenBank: CP020102.1. The Illumina reads were aligned to the referenced genomes using the Burrows-Wheeler short-read alignment tool, BWA version 0.7.9 (11).

Poor sequence coverage resulted in some MA lines being eliminated. Lines with identical mutations occur because of cross-contamination during streaking or because mutations appeared during the growth of the founder colony. If two lines shared over 50% of their mutations then one of the lines was dropped from further analysis. If lines shared less than 50%, each shared mutation was assigned to one of the lines and dropped from the other. If lineage could be established the shared mutation was assigned accordingly, otherwise the mutation was assigned randomly.

**RNA sequencing.** *E. coli* strain PFM144, which is PFM2 Δ*mutL* (10), was grown in LB and aliquots collected during lag (OD = 0.022), log (OD = 0.3), and stationary (OD = 1.5) phase. The number of cells collected was kept constant for each growth phase. Cells were pelleted at 10,000g for 10 min, 1ml of medium was added and the cells were pelleted again at 10,000g for 2 min. The pellets were then flash frozen in liquid nitrogen and stored at -80°. Three biological triplicates were prepared for each growth phase.

RNA was extracted using FastRNA Pro Blue kit (MP Biomedicals). DNA was removed with TurboDNase (Ambion), and confirmed with diagnostic PCR using primer pair “fis forward” and “fis reverse” (Table S3 in the supplemental material). rRNA was removed using RiboMinus magnetic beads kit (Invitrogen). RNA concentration and purity was assessed with an Epoch Microplate Spectrophotometer (BioTek Instruments, Inc.). Libraries were made by the Indiana University CGB and were sequenced at UT Health Science Center on Illumina HiSeq 2500 platform.

**References**

1. Lee H, Popodi E, Tang H, Foster PL. 2012. Rate and molecular spectrum of spontaneous mutations in the bacterium *Escherichia coli* as determined by whole-genome sequencing. Proc Natl Acad Sci USA 109:E2774-E2783.

2. Dewitt SK, Adelberg EA. 1962. The occurrence of a genetic transposition in a strain of *Escherichia coli*. Genetics 47:577-85.

3. Baba T, Ara T, Hasegawa M, Takai Y, Okumura Y, Baba M, Datsenko KA, Tomita M, Wanner BL, Mori H. 2006. Construction of *Escherichia coli* K-12 in-frame, single-gene knockout mutants: the Keio collection. Mol Syst Biol 2:2006.

4. Miller JH. 1972. Experiments in molecular genetics. Cold Spring Harbor Laboratory Press, Cold Spring Harbor, N.Y.

5. Datsenko KA, Wanner BL. 2000. One-step inactivation of chromosomal genes in *Escherichia coli* K-12 using PCR products. Proc Natl Acad Sci USA 97:6640-6645.

6. Miller JH. 1992. A short course in bacterial genetics: a laboratory manual and handbook for *Escherichia coli* and related bacteria. Cold Spring Harbor Laboratory Press, Cold Spring Harbor.

7. Foster PL. 2006. Methods for determining spontaneous mutation rates. Methods Enzymol 409:195-213.

8. Hall BM, Ma CX, Liang P, Singh KK. 2009. Fluctuation analysis CalculatOR: a web tool for the determination of mutation rate using Luria-Delbruck fluctuation analysis. Bioinformatics 25:1564-1565.

9. Foster PL, Lee H, Popodi E, Townes JP, Tang H. 2015. Determinants of spontaneous mutation in the bacterium *Escherichia coli* as revealed by whole-genome sequencing. Proc Natl Acad Sci U S A 112:E5990-E599.

10. Foster PL, Niccum BA, Popodi E, Townes JP, Lee H, MohammedIsmail W, Tang H. 2018. Determinants of base-pair substitution patterns revealed by whole-genome sequencing of DNA mismatch repair defective *Escherichia coli*. Genetics 209:1029-1042.

11. Li H, Durbin R. 2009. Fast and accurate short read alignment with Burrows-Wheeler transform. Bioinformatics 25:1754-1760.
