# Supplementary material for: The Symmetrical Wave Pattern of Base-Pair Substitution Rates across the Escherichia coli Chromosome Has Multiple Causes
Source: mBio. 2019 Jul 2;10(4):e01226-19. doi: 10.1128/mBio.01226-19 (PMC6606806; doi:10.1128/mBio.01226-19)
Supplement: TABLE S2 [file mBio.01226-19-st002.docx]

| **Table S2. Oligonucleotides used in this study** | | | |
| --- | --- | --- | --- |
| Relevant gene | Name | Sequence | Reference |
| *mutL* | mutL Fw | 5′-GCCTGCGCAATTACTTCCTTG-3′ | (1) |
|  | mutL Rv | 5′-CGCAGCTCAATGGCTAACGC-3′ |  |
| *mutS* | mutS FW | 5'-CACGAGAGATACGCTTGCC-3' | This study |
|  | mutS RV | 5'-TCGTGGTTGCCTTTAACCGA-3' |  |
| *oriC* | mioC_2958 FW | 5'-CAAATAAGTATACAGATCGTG-3' | This study |
|  | oriC dwnstrmRV | 5'-CTTTGTCGGCTTGAGAAAGAC-3' |  |
| *oriZ* | yahL1146FW | 5'-GAGATGATGCGCGATTAAATG-3' | This study |
|  | yahMupstrmRV | 5'-CCCCGTACCATACAACAATC-3' |  |
| *rnhA* | rnhA_dwnstrm RV | 5'-CATCGGATTTAGCGTTAAAG-3' | This study |
|  | rnhA_dwnstrm FW | 5'-CTTTAACGCTAAATCCGATG-3' |  |
| *seqA* | seqAFW | 5'-CGAGTTTGTGCGTCCGATTC-3' | This study |
|  | seqARV | 5'-GATGGCATTGGAAACGGCAG-3' |  |
| *tus* | tus FW | 5'-CTCGTGCAAGGCCATTATGC-3' | This study |
|  | tus RV | 5'-AGTTTGACAGCTGGGTACGG-3' |  |
| *matP* | matP FW | 5'-ACAATACTCCTGGCATGGGC-3' | This study |
|  | matP RV | 5'-TGACGCTTACAACCAGGGTC-3' |  |
| *recA* | recAF0102 | 5'-GCATTGCAGACCTTGTGGC-3' | This study |
|  | recAR1134 | 5'-CGACGGGATGTTGATTCTG-3' |  |
| *rnhA* | rnhA_dwnstrm RV | 5'-CATCGGATTTAGCGTTAAAG-3' | This study |
|  | rnhA_dwnstrm FW | 5'-CTTTAACGCTAAATCCGATG-3' |  |
| *recD* | recD_RV | 5'-GACCACGCGCAGTATTCACT-3' | This study |
|  | recD_FW | 5'-GGGTGAAGACAGTTCGGCTT-3' |  |
| *recB* | recB_RV | 5'-GTTCATCTCCCCTGCTGACC-3' | This study |
|  | recB_FW | 5'-CGTTTTTCCCAACCGCAGAG-3' |  |
| *fis* | fisRV | 5'-CTTTTTCAGAACGCGGTGGC-3' | This study |
|  | fisFW | 5'-GGAACACGCTCCAAATGACC-3' |  |
| *hupA* | hupAFW | 5'-CAGGCTGGTCGCGAAATGAG-3' | This study |
|  | hupARV | 5'-GCTAAACACGGCAAGCAGATG-3' |  |
| *hupB* | hupBFW | 5'-CTGTGAAGCGCATTGAGGAAG-3' | This study |
|  | hupBRV | 5'-CTTGAGCACGAGACTGTTTGC-3' |  |
| *dps* | dpsFW_G | 5'-GGGGTCTACGCTGACAGTAC-3' | This study |
|  | dpsRV_BAN | 5'-GATGCACCATTCTGGGGCACC-3' |  |
| *hns* | hnsRV_G | 5'-GTGGTAGAAAAACCGAAAGC-3' | This study |
|  | hnsFW_G | 5'-CATGAATCAGGAAGTTTTAAC-3' |  |
| *rep* | repRv | 5'-CTGTTGACCTTGACGCTTCC-3' | This study |
|  | repFw | 5'-CTTTGGCCCAACGAATCTGC-3' |  |

**References**

1. Lee H, Popodi E, Tang H, Foster PL. 2012. Rate and molecular spectrum of spontaneous mutations in the bacterium *Escherichia coli* as determined by whole-genome sequencing. Proc Natl Acad Sci USA 109:E2774-E2783.
